# Is There Still a Role for Invasive Assessment of Aortic Gradient?

**DOI:** 10.3390/diagnostics13101698

**Published:** 2023-05-11

**Authors:** Domenico Angellotti, Maddalena Immobile Molaro, Fiorenzo Simonetti, Federica Ilardi, Domenico Simone Castiello, Andrea Mariani, Rachele Manzo, Marisa Avvedimento, Attilio Leone, Dalila Nappa, Raffaele Piccolo, Maria Angela Losi, Anna Franzone, Giovanni Esposito

**Affiliations:** Department of Advanced Biomedical Sciences, Division of Cardiology, University of Naples Federico II, 80131 Naples, Italy; dom.angellotti@gmail.com (D.A.);

**Keywords:** aortic stenosis, invasive, gradient, cardiac catheterization

## Abstract

Advances in technology and imaging have expanded the range of tools for diagnosing aortic stenosis (AS). The accurate assessment of aortic valve area and mean pressure gradient is crucial to determine which patients are appropriate candidates for aortic valve replacement. Nowadays, these values can be obtained noninvasively or invasively, with similar results. Contrariwise, in the past, cardiac catheterization played a major role in the evaluation of AS severity. In this review, we will discuss the historical role of the invasive assessment of AS. Moreover, we will specifically focus on tips and tricks for properly performing cardiac catheterization in patients with AS. We will also elucidate the role of invasive methods in current clinical practice and their additional value to the information provided through non-invasive techniques.

## 1. Temporal Trends in Adoption of Diagnostic Tools for the Assessment of Aortic Stenosis

Aortic stenosis (AS) is an increasingly prevalent condition in developed countries. Its aetiology is most commonly degenerative but includes other causes [1]. Regardless of aetiology, stenosis of the aortic valve causes an obstruction of the blood flow from the left ventricle (LV) to the aorta, which generates a systolic flow-dependent pressure gradient across the valve and chronic overload of the LV. In the 1960s, cardiac catheterization emerged as the gold standard for hemodynamic evaluation of valve heart disease. However, in the 1980s, two-dimensional (2D) and Doppler echocardiography was validated as a non-invasive method to provide essential information for AS diagnosis and then became the standard method to assess the severity of AS. Cardiac catheterization is now indicated exclusively to evaluate a structural heart disease that cannot be fully assessed via echocardiography alone.

## 2. Historical Role of Invasive Assessment of Aortic Stenosis

Over the last several decades, advances in technology and imaging have expanded the range of tools for diagnosing AS. In the past, around 70 years ago, AS was primarily recognized through clinical and phonometric assessment. However, there was a growing consensus on the utility of anticipating the identification of patients suitable for surgery to have a beneficial impact on the prognosis. It was clear that this could not be adequately achieved through clinical examination alone, and objective methods to assess the severity of valve disease were urgently needed. Several physicians proposed measuring the pressure gradient across the aortic valve to facilitate decisions about surgical intervention. In 1950, Limon Lason et al. measured left ventricular and aortic pressures in 17 patients using a catheter that was surgically introduced via the radial artery. The introduction of the percutaneous technique to perform heart catheterization by Seldinger, in 1953, prompted the development of new methods to assess the presence and severity of AS [2].

**Figure 1:**
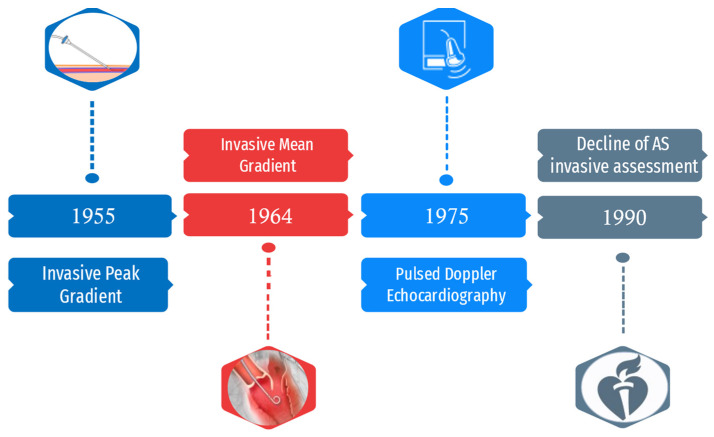
Timeline of the role of invasive assessment in AS diagnosis.

In 1955, Bjork was the first to describe the paravertebral method of left heart catheterization, a technique that he validated in more than 100 cases. Once the left atrium was punctured, a simultaneous pressure measurement in the LV and brachial artery was obtained. A peak pressure gradient of 50 mmHg across the aortic valve in patients with isolated AS was considered an indication for intervention [3]. In 1956, a continuous pressure trace showing a systolic pressure gradient was obtained by withdrawing a catheter from the LV to the aorta. This kind of measurement was obtained in 127 consecutive patients undergoing catheterization through direct left atrial puncture [4]. Later, transthoracic, transbronchial and transseptal approaches were developed to perform this measurement [5]. In another study, Gorman et al. measured the peak systolic gradient obtained from simultaneous left ventricular and brachial arterial pressure recording in 21 patients with clinical diagnosis of severe AS. They observed that a gradient above 45 mmHg was associated with signs of left ventricular strain at ECG and a systolic upstroke duration in the brachial arterial pressure recording of 0.22 s or longer [6]. In 1964, Morgan et al. noted that peak gradient could have been misleading as it was dependent on loading conditions and that mean gradient could have been a better predictor for the severity of stenosis. They also described a method to calculate this new parameter through left heart catheterization. Moreover, they provided a technique for calculation of the estimated valve area with the use of the Gorlin formula. They found dynamically significant AS when the orifice area was reduced to 0.5 cm^2^ or less [7]. In the following years, adjunctive approaches were proposed for the invasive determination of mean gradient as the hemodynamic assessment of AS became a multiparametric evaluation. In 1965, Raftery recorded the pullback of a catheter from LV across the valve via the femoral artery, a method that would soon become the most used for the hemodynamic assessment of AS [8]. However, in view of the morbidity and mortality associated with surgery, several groups stated that hemodynamic data should be used with great caution as their validity was not completely proved. Angiography was another method used to evaluate patients with AS. Grishman et al., in 1947, used venous angiocardiography to assess the presence of left ventricular hypertrophy in patients with AS. Jonsson et al., in 1949, injected contrast medium into the pulmonary artery through a catheter to study the left atrium and LV. Kjellberg et al., in 1955, observed a dome-shaped stenotic aortic valve during ventricular systole. Lehman et al. used transthoracic left ventricular needle puncture to view thickened aortic valve cusps. Odman and Philipson, in 1958, used an arterial retrograde approach to visualize a stenotic aortic valve [2,8]. During the late 1940s, Elder was to the first to perceive the potential of echography as a non-invasive diagnostic tool for heart diseases. However, it took several decades before this technology could be fully applied in cardiology [9]. Thus, 2D echocardiography was introduced in 1974 and pulsed wave (PW) Doppler hemodynamic in 1975. In 1976, it was shown that maximal velocity in the mitral jet could have been measured noninvasively via ultrasonic Doppler using the Bernoulli equation [10]. Shortly after, the same method was used in 41 patients with AS. The data obtained were validated with simultaneous left heart catheterization, and a good correlation was found [11]. This was a paradigm shift in the field of the diagnosis of heart valve disease: utilization of the Doppler matured in many non-invasive laboratories around the world, and invasive assessment of aortic pressures no longer seemed necessary. The severity of AS, associated valve lesions and left ventricular function could now be fully assessed via 2D and Doppler echocardiography, and it could be accomplished serially with a frequency that was not justifiable when only hemodynamic invasive assessment was available. Moreover, several authors proved that data from cardiac catheterization rarely influence patient management. Otto and Pearlman reported that the use of Doppler echocardiography compared to invasive hemodynamic study was a cost-effective approach. Complications during cardiac catheterization occurred in 6.9% of patients [12]. Advances in echocardiography in the late 1980s and the introduction of transoesophageal echocardiography, both for preoperative and intraoperative evaluation of patients with heart valve disease, encouraged clinicians to utilize non-invasive preoperative evaluation even more frequently. Numerous groups who compared echocardiography and cardiac catheterization concluded that invasive hemodynamic measurements were no more routinely necessary [13]. In 1990, the American College of Cardiology/American Heart Association guidelines for the clinical application of echocardiography were published, with the recommendation that selected patients may undergo definitive surgical therapy without the need for cardiac catheterization [14]. Figure 1 summarizes the milestones in the adoption and decline of invasive assessment of AS. It has taken time for changes in clinical practice to occur. In a paper that examined time-related trends in the preoperative assessment of AS, Popovic et al. found that measurement of invasive hemodynamic assessment markedly decreased over time, from 64% in 1986 to 30% in 1994. The performance of invasive hemodynamic assessment during catheterization further decreased during the following decade [15].

## 3. How to Perform an Invasive Assessment of Aortic Stenosis

Over the last few decades, the growing emergence of structural heart disease interventions was accompanied by a renewed interest in the hemodynamic evaluation of heart valve disease. In particular, because of the increased awareness and treatment of severe AS, the knowledge of accurate methods to invasively assess aortic valve hemodynamics remains of crucial importance.

### 3.1. Methodology

The aortic valve area (AVA) can be calculated with the Gorlin equation [16]:

$$\mathrm{AVA}\;({\mathrm{cm}}^{2}):\;\frac{CO(l/min)/[HR\left(bpm\right)\times SEP\left(mSec\right)]}{44.3\times \surd ∆P(mmHg)}$$

where CO is cardiac output, SEP is the systolic ejection period, K = 44.3 (empirical constant) and ∆P is the mean pressure gradient across the valve.

Alternatively, simplified formulae (Hakki formula) provide quick in-laboratory determinations of AVA. AVA can be accurately estimated as cardiac output (CO) divided by the square root of the LV-Aorta peak-to-peak pressure difference [17]. The quick formulae for valve area differ from the Gorlin formula by 18 ± 13% in patients with bradycardia (<65 beats/min) or tachycardia (>100 beats/min).

**Figure 2:**
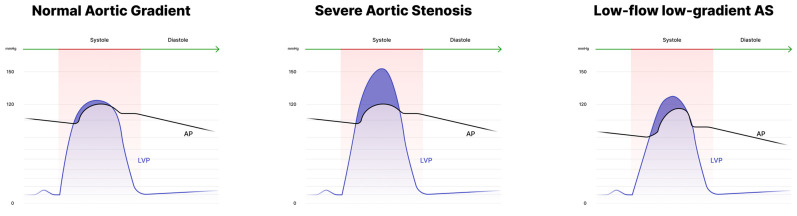
Cardiac catheterization (simultaneous pressure recording) in patients with normal aortic gradient, high-gradient and low-flow low-gradient AS.

These formulae have several limitations: first, they were primarily developed for mitral valve evaluation and later adapted for the aortic valve. Second, accuracy may be lower in patients with bradycardia, tachycardia, aortic regurgitation or low output states. The Gorlin equation, moreover, at low-flow states overestimates the severity of valve stenosis [18]. Irrespective of the used formula, invasive assessment of AS requires accurate measurement of transaortic gradient and CO. Figure 2 displays an estimation of the mean gradient during cardiac catheterization in different conditions.

### 3.2. Technical Aspects

Regardless of the technique, there are many sources of artifacts to keep in mind. The most frequent are: (1) miscalibrated pressure transducers, (2) pressure leaks on catheter manifold or connecting tubing, (3) pressure tubing type, length and connectors, (4) air in the system, (5) catheter sizes (small diameters are prone to artifacts), (6) fluid viscosity (contrast media viscosity tends to damp pressure wave) and (7) position of catheter side holes moving across the aortic valve [19]. Moreover, in patients with critical AS, the catheter across the valve itself may cause further obstruction to outflow and can be a source of erroneous pressure evaluation. This phenomenon is called the Carabello sign and occurs in valve areas of <0.7 cm^2^ when 7 Fr or 8 Fr catheters are used to cross the valve [20,21]. In most cases, crossing the aortic valve is needed to measure the gradient. A transseptal approach should be considered only if crossing the valve is not possible.

Aortic valve crossing requires specific precautions:

- Obtain adequate heparinization according to the patient’s weight;
- Manoeuvre the wire gently to avoid damage to the valve cusps;
- Perform frequent catheter flushing when the guidewire is removed;
- After valve crossing and during catheter exchanges, be careful to guidewire position in the ventricle to avoid perforation.

**Figure 3:**
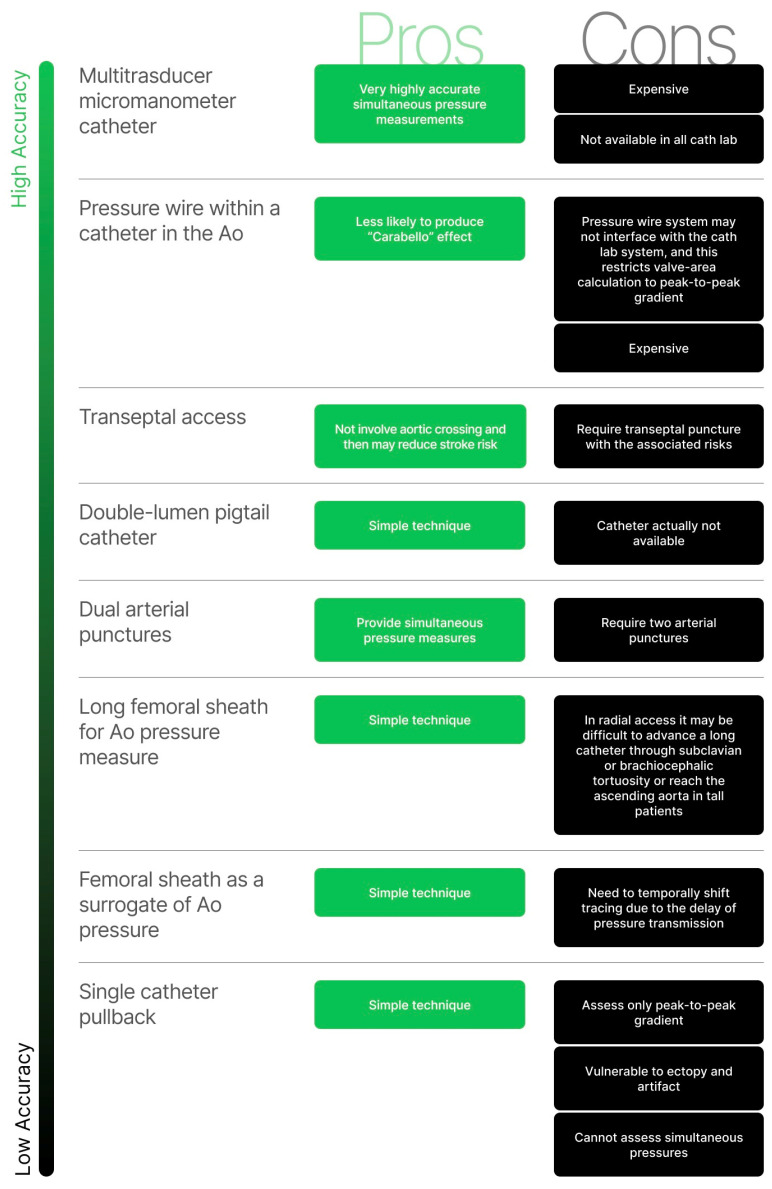
Invasive tools to estimate transaortic gradient. Ao: aorta.

There are several methods utilized to perform a transaortic gradient measurement (Figure 3), each with pros and cons. Initial technique selection is a matter of operator choice and experience.

#### 3.2.1. Single Catheter Left Ventricle Aortic Pullback

This approach consists of advancing a single catheter across the aortic valve; the guidewire is then removed, and generous catheter flushing is performed. The catheter is pulled back into the aorta while continuously recording the pressure curves. Mean transaortic gradient is approximated by superposing the two curves of phenomena that occurred at different times. This technique is simple to perform but can only assess peak-to-peak gradient; it is particularly vulnerable to arrhythmia and artifacts, and it cannot assess simultaneous pressures [22].

#### 3.2.2. Use of Femoral Sheath Pressure as a Surrogate for Central Aortic Pressure

In this case, a catheter is advanced across the aortic valve while the side port of a femoral sheath serves as a surrogate for central aortic pressure. It is a simple technique but is limited by the need to temporally shift tracing due to the inherent delay of pressure transmission between the LV and femoral artery, which tend to over- or underestimate the severity of AS.

#### 3.2.3. Use of Long Femoral Sheath for Measurements of Central Aortic Pressure

An alternative to the abovementioned techniques is to place a 90 cm sheath via the radial or femoral artery, ideally with the distal tip in the ascending aorta. Then, a catheter is advanced across the aortic valve to obtain LV pressure. This method requires using two French-sized smaller catheters than the sheath (for example, a 4 Fr pigtail requires a minimum sheath size of 6 Fr). It is technically simple but, when radial access is performed, it may be difficult to advance a long catheter through subclavian or brachiocephalic tortuosity or reach the ascending aorta in tall patients.

#### 3.2.4. Dual Arterial Punctures

In this case, two arteries are involved in the puncture. When the two accesses are obtained, and the two sheaths are placed, a catheter is advanced through one sheath into the ascending aorta and the other one is advanced through the second sheath across the aortic valve. At this time, it is possible to record left ventricular and aortic pressures simultaneously. This technique has the advantage of providing simultaneous pressure but requires the involvement of two arteries and, thus, increases the risk of complications.

#### 3.2.5. Double-Lumen Pigtail Catheter

The double-lumen pigtail catheter was the most widely used invasive technique for over a decade. This specific catheter has a 6 Fr outer diameter and a 4 Fr inner lumen, allowing for simultaneous measurement of left ventricular and aortic pressures [23]. Unfortunately, the catheter was recalled from the market due to several safety issues [24].

#### 3.2.6. Mother and Daughter Technique

This was a technique developed as an alternative to dual-lumen catheters. A 4 Fr 110 or 120 cm pigtail was inserted into a 6 Fr 90 cm guide catheter. Thus, a dual-lumen, homemade catheter was developed with the use of a detachable valve or a homemade equivalent such as a trimmed 4 Fr sheath [25]. The cons of this technique are that not all cath-labs have a full range of 4 Fr catheters, 90 cm guiding catheters and detachable valves.

#### 3.2.7. Transeptal Access

In most patients, hemodynamic assessment of the LV can be performed with retrograde aortic catheterization. However, in a small subset of patients (e.g., those with hypertrophic cardiomyopathy), optimal hemodynamic assessment cannot be performed with standard access but requires transeptal access. This technique is at a higher risk for major complications (i.e., cardiac perforation, puncture into the aortic root, pericardial tamponade and embolus from the left atria), and it should not be performed in patients that cannot maintain supine position, with left or right atrial thrombus, atrial myxoma or absence of right femoral venous access to the right atria as a result of masses, thrombus or other causes of obstruction. Adjunctive imaging with either transoesophageal or intracardiac echocardiography has been increasingly used to improve the safety and accuracy of the transeptal puncture. After the puncture, a pigtail or a balloon wedge catheter is advanced in the LV. Then, another arterial access point is obtained to advance a pigtail catheter into the ascending aorta. Thus, simultaneous pressures are measured.

#### 3.2.8. Pressure Wire within a Catheter in the Ascending Aorta

A catheter (typically 5 Fr) is advanced in the ascending aorta. Then, a Tuohy valve is placed at the back of the catheter. A pressure guidewire is advanced through the catheter, and pressures between the catheter and pressure wire are equalized. At this time, the aortic valve is crossed conventionally with a 0.035” guide. The guide is newly exchanged with a pressure wire, which is then advanced into del LV. The catheter is retrieved into the ascending aorta while the pressure wire stays within the ventricle. Simultaneous pressures can be obtained, enabling the assessment of the Hakki-derived valve area [17]. In some cases, a hemodynamic system permits assessment of the mean gradient for the Gorlin-derived valve area. This system is less likely to produce the “Carabello” effect because only a wire and not a catheter is advanced through the stenotic aortic valve [26], but the pressure wire system may not interface with the cath-lab system, and this restricts valve area calculation to peak-to-peak gradient [27,28].

#### 3.2.9. Multi-Transducer Micromanometer Catheter

This type of catheter provides highly accurate simultaneous pressure measurements, but its cost is prohibitive and, therefore, it is rarely used.

## 4. Current Role of Invasive Assessment of Aortic Stenosis

Calcific degeneration of the tricuspid or bicuspid aortic valve and rheumatic disease are the most observed causes of valvular AS. Echocardiography is the diagnostic tool of choice to assess the aortic stenosis aetiology since it allows for the evaluation of valve morphology. Moreover, ultrasound imaging together with integrated Doppler allows for the determination of the level of obstruction based on the site of the increased velocity: sub-valvular, valvular or supravalvular AS [12]. Cardiac computed tomography could be useful to confirm the findings on transthoracic echocardiography (TTE): it is able to determine an aortic valve calcification score, whereby a high score may indicate severe AS, particularly in patients with degenerative aetiology. Furthermore, it allows one to assess the valve anatomy in greater detail. Invasive assessment of AS is not influenced by specific aetiologies. Understanding the haemodynamic principles behind AS pathophysiology allows one to critically integrate the data provided from non-invasive diagnostic techniques and to acknowledge the still-important role of invasive assessment.

Invasive assessment of AS is useful in the following conditions:

1. Inconsistency among non-invasive measurements;
2. Evaluation of special subsets of AS, including low-flow, low-gradient AS;
3. Identification of predictors of procedural and clinical outcomes in patients undergoing Transcatheter Aortic Valve Implantation (TAVI).

Inconsistent grading is more prevalent with the non-invasive assessment of aortic valve stenosis than with cardiac catheterization [29]. Errors with the continuity equation may arise from its assumption of a circular left ventricular outflow tract (LVOT), particularly in elderly patients [30]. However, only when data from non-invasive testing remain inconclusive, especially if there is discordance in the degree of severity by physical examination and non-invasive tests, catheterization can be helpful to determine the severity of AS. Omran et al. found that patients with valvular AS who undergo retrograde catheterization of the aortic valve have a substantial risk of clinically apparent cerebral embolism, and they frequently have silent ischaemic brain lesions proven by magnetic resonance imaging of the brain [31]. These data support the premise that routine crossing of the aortic valve in AS patients should be avoided when the severity of the AS has already been adequately defined through non-invasive techniques. Currently, 2D TTE is the gold-standard tool for AS assessment but may be inaccurate in the following settings: poor image quality, low flow states and/or heart rhythm disorders. Echocardiography is highly operator-dependent: a misalignment between the Doppler beam and the direction of the aortic jet can result in underestimation of the pressure drop and, on the other hand, the pressure drop may be overestimated in severe anaemia or conditions associated with high output (Table 1). Invasive assessment of doubtful AS by measuring simultaneous transaortic gradient using a pressure wire may be a useful technique in a large proportion of patients. A study conducted by Chopard et al. showed that there was agreement for severe aortic stenosis, defined by an AVA < 0.6 cm^2^/m^2^ between the simultaneous method (SM) and 2D-TTE and between SM and the pullback method (PM) but poor agreement between 2D-TTE and PM [32]. These findings are in line with those previously reported by Brogan et al., who found that PM may yield an inaccurate assessment of mean transaortic gradient in cases of low transvalvular flow, whatever the cardiac rhythm is [33]. Patients with severely reduced LV systolic function may present with low stroke volume and low gradient: in this setting, ensuring that the small, calculated AVA is due to true severe AS or “pseudo-aortic stenosis” could be challenging. In the latter, the aortic valve is moderately stenotic, but the leaflet opening is poor due to a low CO. However, in low-flow state conditions, rest cardiac catheterization does not provide any additional information on stenosis severity or LV function besides that obtained from rest echocardiography. Dobutamine stress echocardiography (DSE) is the gold standard to differentiate true AS from pseudosevere AS and assess the presence of the LV flow reserve, but it is also subject to technical pitfalls and measurement errors. In true AS, ∆P mean rises to >40 mmHg. If CO becomes normal but ∆P mean stays low (<30 mmHg), with an increase in AVA, then pseudostenosis is present. Some patients are not able to increase CO due to a reduced contractile reserve (increase in stroke volume < 20%). These patients have indeterminate AS and require a multiparametric evaluation, considering that the long-term prognosis after valve intervention worsens in these patients [34]. A poorer correlation between the invasive and non-invasive assessment has been reported in elderly patients, a population in which a low-flow status might easily arise. In particular, invasive methods underestimated AVA compared with Echo in a cohort of elderly patients being considered for aortic valve replacement. Lower cut offs for severe AS disease may be used for invasive measurement of AVA in this population to prevent the risk of overestimating stenosis severity, an error that could have a dismal impact on patient management and prognosis [30]. Despite patients with low-gradient severe AS generally have worse prognosis and higher mortality [35], some studies suggest a survival benefit of TAVI in patients with true severe low-gradient AS. A significant functional improvement was observed at 1-year follow-up among low-flow low-gradient patients after TAVI [36]. Furthermore, TAVI is associated with less prosthesis–patient mismatch, which has been shown to be highly detrimental in patients with reduced LVEF, including those with classical low flow-low gradient AS. Early recognition and correct diagnosis of a patient with low-gradient AS is crucial to improve their mortality and morbidity. In these patients, invasive assessment with dobutamine infusion could be helpful to normalize CO. Conversely, in patients with low gradient and preserved ejection fraction, lowering of the peripheral resistance with nitroprusside should lead to an increase in aortic valve gradient and change in valve area. Lloyd et al. highlighted the utility of nitroprusside in assessing patients with low-gradient aortic stenosis by neutralizing the effects of arterial afterload on myocardial function and allowing for a more targeted assessment of aortic valve disease [37]. Patients with AS often have a high prevalence of coronary artery disease (CAD) [38]. Although computed tomography angiography is routinely used for preprocedural planning, invasive coronary angiography remains the optimal method for diagnosing CAD in patients with valvular heart disease with a class I, level of evidence C recommendation in the current guidelines [39]. Invasive evaluation of the presence and extent of CAD is also based on the assessment of coronary physiology, with accumulating evidence about the role of these indexes in the setting of severe AS.

Moreover, coronary angiography is the only method that allows for prompt treatment of CAD in the same setting, when necessary. Furthermore, the results of coronary angiography can help guide treatment decisions, such as choosing between surgery and percutaneous treatment. Additionally, pulmonary hypertension (PH) frequently coexists with severe aortic stenosis, and several studies have previously reported a prognostic value of baseline PH in predicting mortality outcomes in patients with severe AS who undergo TAVI [40,41,42]. Postcapillary PH (mean pulmonary artery systolic pressure >20 mmHg and left ventricular end-diastolic pressure >15 mmHg) proved to be benign with regard to post-TAVI outcomes compared to precapillary (mean pulmonary artery systolic pressure >20 mmHg and left ventricular end-diastolic pressure ≤15 mmHg) or combined post- and precapillary PH (mean pulmonary artery systolic pressure >20 mmHg and left ventricular end-diastolic pressure >15mmHg and pulmonary vascular resistance >2 WU) [43,44]. Postcapillary PH is always associated with left heart disease. As expected, this group of patients together with combined PH were the only ones that showed the greatest improvement after TAVI, since the improvement in their valvular problem with the procedure improved their left heart function [45] and subsequently their PH, which was induced by the backward transmission of high left-sided filling pressures. The presence of PH is associated with increased short- and long-term mortality and also with an higher risk of stroke and acute kidney injury after TAVI. According to the hemodynamic presentation, it is important to invasively stratify patient undergoing TAVI according to the PH to predict the acute response to treatment and mortality. In patients with severe AS and LV hypertrophy, it may be expected that diastolic LV dysfunction persists after TAVI, causing persistent postcapillary PH in the majority of patients, and this could be prognostically relevant. After TAVI, pulmonary artery systolic pressure and right ventricle function improved in patients with postcapillary hypertension but not in those with precapillary hypertension. Right heart catheterization remains the gold standard for the accurate diagnosis of PH; however, its routine use of prior TAVI is questionable in predicting mid-term clinical outcomes [46].

## 5. Invasive-Assessment-Related Complications

Invasive assessment of AS inherits the risk of complications, with the major ones occurring in less than 1% and mortality in 0.05% of cases. Among the minor complications, the more frequent are those relative to the access site, such as vascular injury and bleeding. Moreover, risks related to ionizing radiation exposure should not be underestimated.

Among major complications, concern has been raised about cerebral embolization: it results from aortic root manipulation and retrograde aortic valve crossing, leading to debris migration. In a study by Omran et al., 22% of patients undergoing retrograde catheterization showed silent cerebral infarction at magnetic resonance imaging, while 3% suffered clinically apparent neurological deficits [31]. Notably, elderly patients with AS have a high prevalence of tortuosity in the subclavian and brachiocephalic region. This can make retrograde crossing of the aortic valve through radial artery technically difficult, likely requiring more attempts and longer procedure time. Consequently, the risk of cerebrovascular events may increase in elderly patients, since the time required to cross the aortic valve has been found to be the most important independent predictor of silent cerebral infarction [47].

## 6. Conclusions

AS incidence is increasing as it has become one of the most common valvular heart diseases in developed countries. In the past, cardiac catheterization was considered the benchmark for evaluating valvular diseases; afterwards, 2D and Doppler echocardiography was validated as a non-invasive method for AS diagnosis and is now the standard approach for assessing the severity of AS. In the current guidelines, the use of cardiac catheterization is limited to the evaluation of cases that cannot be completely assessed through echocardiography. However, recently, there has been a rise in interventions for structural heart diseases along with a revived focus on its hemodynamic assessment. This renewed interest has been partly driven by the increased awareness and treatment of severe AS, making it important for every cardiological intervention to have confidence with accurate invasive methods for assessing aortic valve hemodynamics.

## Figures and Tables

**Table 1:** Comparison of cardiac catheterization vs. TTE in AS diagnostic pathway.

	Advantages	Pitfalls
***Echocardiography***	Non invasive Widely anatomical assessment Instantaneous transaortic pressure drop measurement	Operator dependent Poor quality of image Lack of alignment Low flow states Hearth rhythm disorders Overstatement in case of high output condition
***Cardiac*** ***Catheterization***	Direct measurement of mean transaortic pressure drop Assessment of hemodynamic status	Invasive Radiation exposure Risk of embolic stroke Unknown exact anatomic point where pressure recovery occurs

## Data Availability

All data underlying this article will be shared on reasonable request to the corresponding author.
